# Radiation Curing of Phosphorus Telomer-Based Coatings Using UV LEDs or Medium-Pressure Mercury Lamp

**DOI:** 10.3390/ma16237493

**Published:** 2023-12-04

**Authors:** Agata Kraśkiewicz, Agnieszka Kowalczyk

**Affiliations:** Department of Chemical Organic Technology and Polymeric Materials, Faculty of Chemical Technology and Engineering, West Pomeranian University of Technology in Szczecin, 70-322 Szczecin, Poland; agata.kraskiewicz@zut.edu.pl

**Keywords:** phosphorus-containing coatings, telomerization, UV curing, (meth)acrylates, LED

## Abstract

In the presented study, UV LEDs (365 nm) or a medium-pressure mercury lamp (UV-ABC) were verified as UV radiation sources initiating the photocrosslinking process of varnishes based on novel photopolymerizable phosphorus (meth)acrylate oligomers. Coating formulations were composed of (meth)acrylic/styrene telomers with terminal P-atoms (prepared via a UV phototelomerization process) and different photoinitiators (HAPs, APOs, or APO blends). The kinetics of the UV crosslinking process of the coating formulations depending on UV irradiation and the UV range was investigated by the photo-DSC method. Moreover, the hardness of the varnishes and the conversion of double bonds using the FTIR method were tested. The photopolymerization rate and the photoinitiation index, depending on the type of photoinitiator, were as follows: APOs < APO blends < HAPs. However, the highest coating hardness results were obtained using the least reactive photoinitiator from the APO group, i.e., Omnirad TPOL, or a mixture of three different types of acylphosphine (Omnirad BL 750). The greater effectiveness of the above-mentioned APOs over HAP was also demonstrated when using a UV LED lamp at 365 nm with a low UV dose and UV irradiance, thanks to the presence of phosphoric acid diester in the coating composition, acting as both a telogen and an antioxidant.

## 1. Introduction

UV radiation curing is becoming increasingly appreciated at present. The growing interest in this technology is related to its ecological and economic benefits, such as high process rates at room temperature, low volatile organic compound (VOC) emissions, and the use of solvent-free formulations [1,2,3,4]. The application areas of UV-curable materials include electronics [5], aerospace [6], packaging [7], and the wood industry [8], as well as medicine [9] and dentistry [10].

The most common radiation sources used in UV curing technology are medium-pressure mercury lamps. Their popularity is mainly due to the emission of three types of UV radiation (UV-A, UV-B, and UV-C) and relatively low prices. However, some limitations are also noticeable, e.g., the production of harmful ozone, toxic mercury content, a short lifetime (approximately 1500–2000 h), high energy consumption, and an intensive temperature rise [11,12,13]. In addition, it is important to take into account that the EU has imposed restrictions on the production of mercury-containing lamps from 2027 [14]. For these reasons, promising sources of UV radiation are UV-LEDs. The longer lifetime, elimination of mercury, and no ozone generation make them inherently environmentally friendly. In addition, both their 50% lower energy consumption (due to the possibility of immediate switching on/off or their use only when necessary, and their narrow wavelength distribution) and their suitability for heat-sensitive materials further encourage the replacement of UV mercury lamps with UV LED systems [15,16,17].

Typical photopolymerizable coating formulations are composed of multifunctional monomers or/and oligomers, photoinitiator(s), and reactive diluents [18]. The type and concentration of ingredients depend on the desired properties of the final products. (Meth)acrylate-functionalized oligomers and monomers are frequently used due to their high reactivity and thus wide modification possibilities [19]. In this context, particularly interesting are phosphate (meth)acrylates and poly(meth)acrylates—they allow the formation of coatings marked by unique properties, e.g., good adhesion to steel substrates [20], corrosion resistance [21], fire resistance [22], and antimicrobial resistance [23].

Syntheses of (meth)acrylate copolymers bearing functional groups—for example, with phosphorus atoms—have been carried out mainly via free radical polymerization techniques (or even via photopolymerization) [24]. It is also worth mentioning that the preparation of oligomers/polymers suitable for specific applications (well-defined copolymers) requires conducting controlled polymerization, e.g., atom transfer radical polymerization (ATRP) [25], reversible addition–fragmentation transfer process (RAFT) [26], or nitroxide-mediated radical polymerization (NMP) [27].

However, a less frequently described method of preparation for copolymers with controlled structures is telomerization—a special type of polymerization consisting of a chain reaction between a telogen (also called a chain transfer agent, a molecule with easily radically cleavable bonds, e.g., P-H, C-Br, S-H) and one or more polymerizable compounds (taxogens/monomers, exhibiting ethylenic unsaturation), which leads to the synthesis of low-molecular-weight oligomers with terminal groups derived from telogen [28,29]. Regarding the synthesis of phosphorus-containing oligomers by telomerization, dialkyl phosphites, phosphorus acid, phosphorus tri- and pentachloride, and alkyl dichlorophosphines or dichlorophosphonates are typically used as telogens. However, phosphate esters (e.g., dimethyl phosphate—proposed in these studies) are described in the literature reports as less effective chain transfer agents. Furthermore, the majority of reported telomerization reactions involving organophosphorus compounds are thermally initiated [30]. As stated, the preparation of phosphorus-containing telomers (oligomers) by UV-induced telomerization has not been noted thus far (apart from the work of authors [31]). The novelty of this type of coating is that they are obtained in an environmentally friendly way (without the use of organic solvents, with low demand for electricity and quickly). Photochemically induced telomerization allows the preparation of (semi)telechelic oligomers containing P-atoms in their structure (P-telomers). The authors’ previous works presented in detail a new method of obtaining acrylate oligomers containing phosphorus, a comparison to other preparation methods, and the specific properties of the coatings obtained from these P-telomers [32].

In this study, the influence of the type of radiation source (medium-pressure mercury lamp or UV LEDs) and the photoinitiator (acylophosphine oxides or α-hydroxyalkylphenones) on the UV curing process, as well as the hardness of coatings based on phosphorus-containing (meth)acrylic resins (e.g., P-telomer syrups), were investigated.

## 2. Materials and Methods

### 2.1. Materials

The (meth)acrylic syrup containing phosphorus telomers (the P-telomer syrup, P-TS) was synthesized using the following monomers (a–d), P-telogen (e), and radical photoinitiator (f):

(a) n-butyl acrylate (BA; BASF, Ludwigshafen, Germany);
(b) methyl methacrylate (MMA; Sigma Aldrich, Steinheim, Germany);
(c) 2-hydroxyethyl acrylate (HEA; Across Organics, Geel, Belgium);
(d) styrene (STY; Sigma Aldrich, Steinheim, Germany);
(e) dimethyl phosphite (DMPh, Sigma Aldrich, Steinheim, Germany);
(f) bis(2,4,6-trimethylbenzoyl)-phenylphosphineoxide (Omnirad 819, IGM Resins, Waalwijk, The Netherlands).

The UV-curable varnish compositions were prepared using the P-telomer syrup (P-TS) and commercial type I radical UV photoinitiators (IGM Resins, Waalwijk, The Netherlands).

(i) α-Hydroxyalkylphenones (HAPs):
  2-hydroxy-1-[4-[4-(2-hydroxy-2-methylpropionyl)benzyl)phenyl)-2-methylpropan-1-one (Omnirad 127);
  1-hydroxycyclohexylphenyl ketone (Omnirad 184).
(ii) Acylphosphine oxides (APOs):
  - 2,4,6-trimethylbenzoyl-diphenyl phosphine oxide (Omnirad TPO);
  - ethyl(2,4,6-trimethylbenzoyl)-phenyl phosphinate (Omnirad TPO-L);
  - bis(2,4,6-trimethylbenzoyl)-phenylphosphine oxide (Omnirad 819).
(iii) Blends of APOs:
  - a blend of bis(2,4,6-trimethylbenzoyl)-phenylphosphine oxide (ca. 95 wt%) and ethyl(2,4,6-trimethylbenzoyl)-phenyl phosphinate (ca. 5 wt%) (Omnirad 2100);
  - a blend of ethyl(2,4,6-trimethylbenzoyl)-phenyl phosphinate (ca. 60 wt%), 2,4,6-trimethylbenzoyl-diphenyl phosphine oxide (ca. 20 wt%), and bis(2,4,6-trimethylbenzoyl)-phenylphosphine oxide (ca. 20 wt%) (Omnirad BL 750).

**Figure 1:**
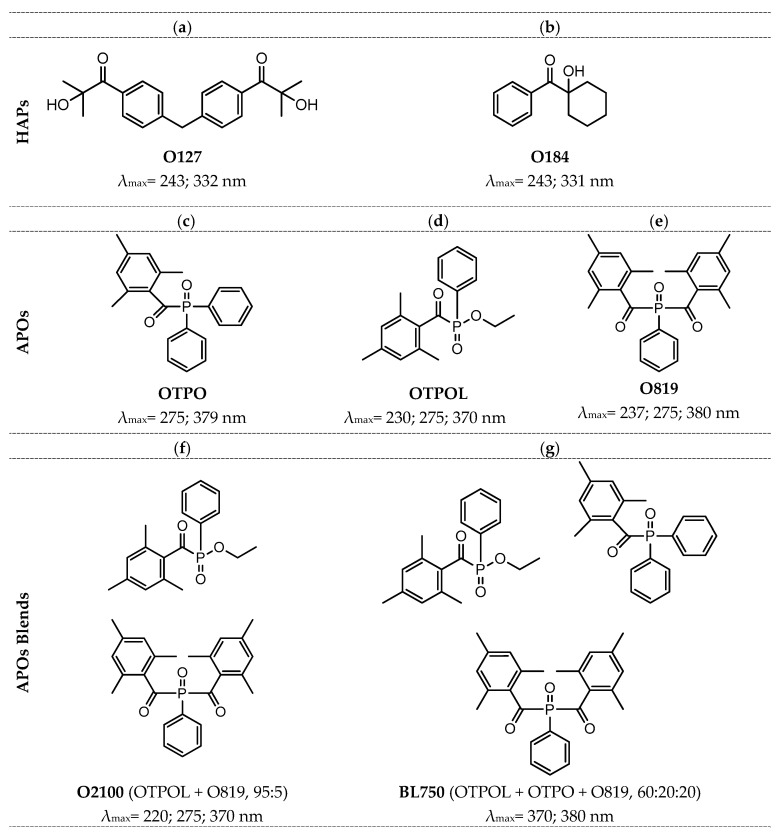
Chemical structures of the tested PIs: Omnirad 127 (**a**), Omnirad 184 (**b**), Omnirad TPO (**c**), Omnirad TPOL (**d**), Omnirad 819 (**e**), Omnirad 2100 (**f**), Omnirad BL750 (**g**).

The chemical structures of the tested photoinitiators (PIs) are shown in Figure 1.

### 2.2. Preparation of the P-Telomer Syrup, Varnish Compositions, and Coatings

**Figure 2:**
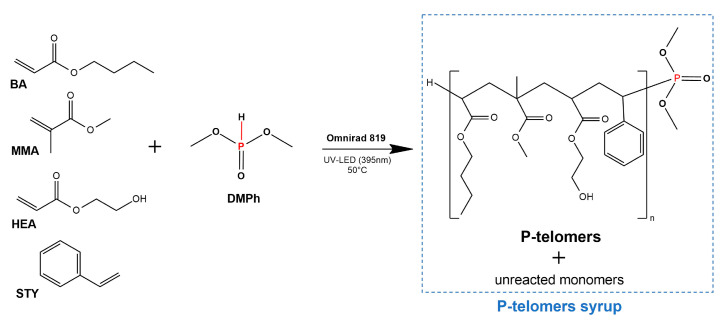
Phototelomerization process of BA, MMA, HEA, and STY monomers with DPMh as P-telogen.

The P-telomer syrup (P-TS) was synthesized via the UV-induced telomerization (phototelomerization) process of the monomer mixture (BA, MMA, HEA, STY), the P-telogen (DMPh), and the radical photoinitiator (O819). The phototelomerization was carried out at 50 °C for 50 min in a glass reactor (250 mL) equipped with a mechanical stirrer, a water cooler, a thermocouple, and a capillary dosing inert gas (Ar). The UV LED stripe (λ = 390 ± 5 nm; MEiSSA, Warsaw, Poland) was used as a UV light source. The composition of the reaction mixture is shown in Table 1, while the schematic structure of the phototelomerization reaction is shown in Figure 2.

The UV-curable varnish compositions were prepared by the mechanical mixing of the P-TS and the selected radical photoinitiator (HAP, APO, or APO blend). Seven coating compositions were obtained (Table 2).

**Figure 3:**
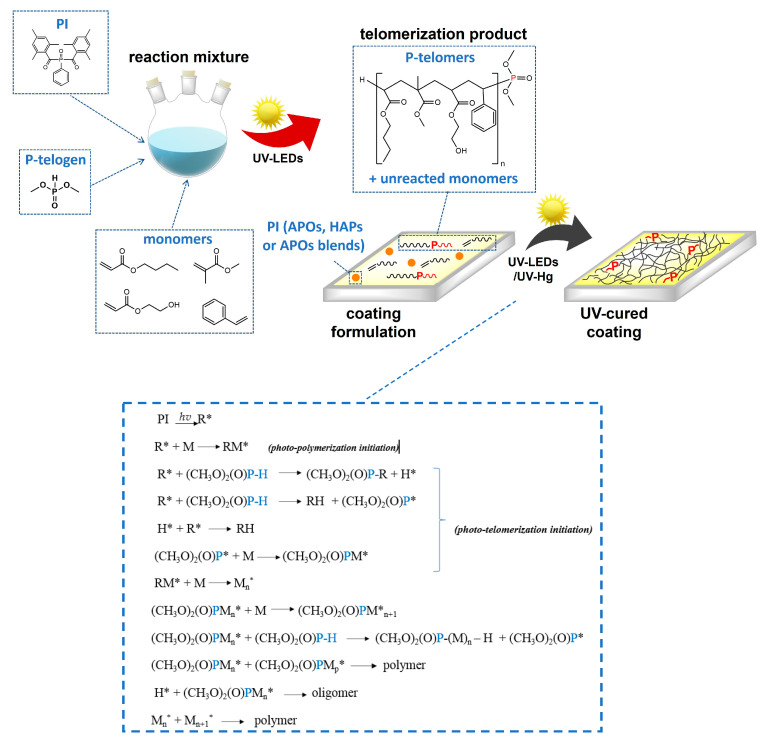
Schematic illustration of the varnish preparation (M—monomer; R—radical, (CH_3_O)_2_PH is P-telogen).

Then, they were stored for 24 h in a dark place and applied onto glass substrates using a gap applicator (60 µm) and UV-irradiated by means of a medium-pressure mercury lamp at 200–420 nm (UV-ABC, Hönle UV-Technology, Gräfelfing, Germany; total UV dose of 6 J/cm^2^) or UV LEDs at 365 nm (own production; UV dose of 6 or 30 J/cm^2^). A schematic illustration of the varnish preparation is given in Figure 3.

### 2.3. Characterization of the P-Telomer Syrup

The P-telomer syrup was characterized in our previous publication (Table 3, [31]). The dynamic viscosity (η) was measured at 25 °C using the DV-II Pro Extra viscometer (spindle #7, 50 rpm; Brookfield, New York, NY, USA). The solid content (SC) was determined with a thermobalance (Radwag, Warsaw, Poland); during the measurement, samples (ca. 2 g) were heated in aluminum pans at 105 °C for 4 h. Monomers and telogen conversions were evaluated by proton nuclear magnetic resonance ^1^H NMR and P NMR (Bruker DPX Avance III HD Spectrometer; 400 MHz); a solution of the P-TS in CDCl_3_ was used. The required parameters were characterized by comparing the intensities of the monomers’ peaks and the internal standard’s (1,3-dinitrobenzene) peaks [32]. Gel permeation chromatography (GPC) was used for the evaluation of the molecular masses (M_w_, M_n_) and polydispersity (PDI) of the dry P-telomers (P-telomer syrup was heated at 140 °C for 4 h before the test to remove unreacted monomers).

### 2.4. Kinetic Studies of UV Curing of the Varnish Composition

The kinetic studies of the photocuring processes, i.e., the reaction rate (*R_p_*, W/g) and photoinitiation index (*I_p_*, s^−2^), were determined using the photo-DSC method (a differential scanning calorimeter with UV equipment; Q100, TA Instruments, New Castle, DE, USA). The samples (varnish compositions, ca. 5 mg) were polymerized in open aluminum pans under isothermal conditions at 25 °C in high-purity nitrogen purged with 50 mL·min^−1^ before and during the polymerization reaction. The polymerization was initiated with a UV lamp in three variants: (1) λ = 365 nm, I_0_ = 90 mW/cm^2^; (2) λ = 250–450 nm, I_0_ = 90 mW/cm^2^; (3) λ = 250–450 nm, I_0_ = 500 mW/cm^2^. The UV polymerization conditions were similar to those of the UV curing process, realized with a UV-ABC-type medium-pressure mercury lamp or UV LEDs. The above-mentioned parameters (*R_p_*, *I_p_*) were calculated according to Equations (1) and (2), respectively. In the calculations, the amounts of unreacted monomers indicated by NMR studies were taken into account.

(1) $$R_{p}=\frac{\left(\frac{dH}{dt}\right)}{m}\left[W/g\right]$$

(2) $$I_{p\;}=\frac{R_{p}^{max}}{t_{max}}\;\left[s^{-2}\right]$$

where *dH*/*dt*—the recorded heat flow during UV irradiation; *m*—the mass of the sample; *R_p_^max^*—the maximum polymerization rate calculated based on the theoretical heat value for the complete degree of conversion; *t_max_*—the time to reach the maximum polymerization rate [31].

### 2.5. Characterization of the Varnishes

The basic mechanical property of the coatings—the pendulum hardness—was determined using a König pendulum (AWS-5, Dozafil, Warsaw, Poland) according to the PN-EN ISO 1522 standard (five measurements for each sample were performed) [33].

The degree of crosslinking of the varnish coatings was checked using a Fourier transform infrared spectroscope with ATR accessories (Nicolet 380, ThermoScientific, Waltham, MA, USA). The conversion of double bonds ($DC_{c=c}$) was calculated according to Equation (3). The DC was evaluated by the comparison of the area of the C=C stretching vibration bands at 1635 cm^−1^ (the absorption bands of νC=C [34]) and at 812 cm^−1^ (bond torsion vibration -C=CH_2_ [35]) with the area of the C=O stretching vibration band at 1724 cm^−1^.

(3) $$DC_{c=c}=\left(1-\frac{\frac{A_{t}^{812}+A_{t}^{1635}}{A_{t}^{1724}}}{\frac{A_{0}^{812}+A_{0}^{1635}}{A_{0}^{1724}}\;}\right)\cdot 100\;\left[\%\right]$$

where *A*_0_ is the initial intensity of the peak at a specific wavenumber and *A_t_* is the intensity of the peak at the same specific wavenumber after the UV curing process.

## 3. Results and Discussion

### 3.1. Kinetic Studies of UV Curing of the Varnish Composition

**Figure 4:**
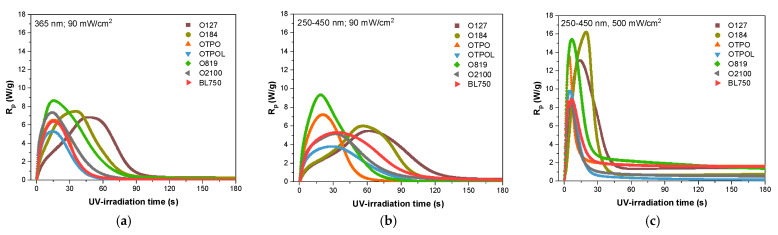
UV curing reaction rates for the varnish compositions in different UV curing conditions: (**a**) 365 nm, I_0_ = 90 mW/cm^2^; (**b**) 250–450 nm, I_0_ = 90 mW/cm^2^; (**c**) 250–450 nm, I_0_ = 500 mW/cm^2^.

The polymerization kinetics of varnish compositions with different PIs and UV curing conditions were followed by photo-DSC. In the study, systems containing HAPs, APOs, or APO blends were compared. It is worth noting that the weight addition of PIs was the same, i.e., 1 wt. part (industry practice was adopted in the study), while the molar concentration varied (about 0.003 mol/100 g of photoreactive mixture for all PIs; in the case of O184, ca 0.004 mol) (Table 2). It should also be mentioned that the photoreactive mixture was a telomeric syrup consisting of approximately 50% (meth)acrylate-styrene oligomers containing terminal phosphorus atoms (SC = 47%). Based on the NMR analysis, DMPh (as P-telogen) was incorporated into the oligomer’s structure in about 30%. The remainder of the DMPh was in the P-TS. Therefore, during the UV irradiation of the coating composition (P-TS + PI), not only the photopolymerization of unreacted monomers but also (to some extent) phototelomerization occurred (Figure 3). Thus, a mixture of unreacted monomers and P-telogen was involved in the photopolymerization (and phototelomerization) process, which consisted of 53% BA, 23% MMA, 19% HEA, and 5% STY (calculated from NMR). The process of creating a poly(meth)acrylate network in the coating takes place in the presence of linear oligomers with terminal P-atoms, which act as steric hindrances. The resulting cured coating is therefore a three-dimensional polymer network consisting of interconnected (entangled) macromolecules (without covalent nodes). The study aimed to demonstrate whether matching the type of photoinitiator in terms of its electromagnetic radiation absorption capacity to the characteristics of the radiation source, i.e., the range of wavelengths emitted, is as important for the photopolymerization process as is commonly believed. A particular case of photopolymerization is considered here, i.e., in the presence of an organophosphorus compound as a chain transfer agent (phototelomerization). As previously mentioned, the exposure of the samples was carried out in three variants in the photo-DSC chamber, reflecting the actual exposure conditions of the varnish—namely, with low UV irradiance (90 mW/cm^2^) and a specific wavelength, i.e., 365 nm or in the broad UV range (250–450 nm) (variants one and two, respectively), and with high UV irradiance and a broad UV range (variant three). The kinetic curves of systems irradiated at low UV irradiance, i.e., 90 mW/cm^2^ (Figure 4a,b), are similar in some respects. Namely, the maximum reaction rates are between 4 and 8 W/g, regardless of the UV range. Only in the case of O184, the *R_p_^max^* value is higher, at approximately 10 W/g (which in turn is caused by the higher molar concentration of this photoinitiator; see Table 2). In contrast, for systems irradiated at high irradiance (500 mW/cm^2^), the R_p_^max^ values are twofold higher (8 to 16 W/g). As can be seen, at high UV irradiance, the photopolymerization process occurs much faster, i.e., approximately 30 s for APOs and APO blends and approximately 45 s for HAPs (Figure 4c).

**Figure 5:**
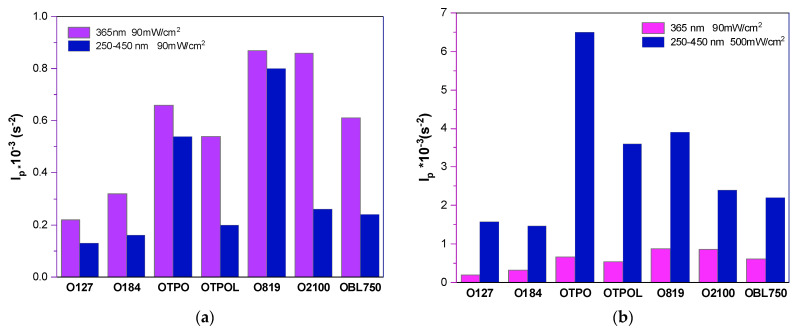
Photoinitiation indices for the varnish compositions depending on PIs and UV curing conditions: (**a**) I_0_ = 90 mW/cm^2^, (**b**) I_0_ = 90 mW/cm^2^ or 500 mW/cm^2^.

This is because, as is well known, the rate of photopolymerization initiation depends on the intensity of absorbed light (I_a_), which corresponds to the number of photons absorbed per unit volume and time. The intensity of the absorbed light (I_a_) depends on the intensity of the incident light (I_0_), i.e., that emitted by the radiation source used, and on the absorbance of the photoinitiator. The latter, in turn, depends on the photoinitiator concentration and the thickness of the layer being exposed. In the results presented here, it can be assumed that the thickness of the irradiated layer is the same for each sample (approximately 5 mg of the sample was fed into standardized aluminum crucibles). To some extent, it can be considered that the molar concentration of the photoinitiator is the same (except for O184). Thus, the kinetic curves considered describe well the kinetics of the process as a function of the incident light intensity (I_0_). The longest photopolymerization times were recorded when irradiated in the broad UV range (250–450 nm) and at low irradiance (90 mW/cm^2^); see Figure 4b. They were approximately 105 to 145 s (for systems with HAPs) and 60 to 120 s (for APOs and blends of APOs), respectively. The HAPs had strong absorption in the short-wave UV range (230–270 nm) and in moderate concentrations. They also had weak absorption at longer wavelengths of up to 360 nm. They are mainly used in clear coatings and produce hard coatings and are less suitable for styrene formulations due to their longer triplet lifetimes. Therefore, the highest reaction rate with a high UV irradiance value was recorded for the system with O184 (Figure 4c). Interestingly, in a wide range of UV radiation and at low irradiance (Figure 4b), the photopolymerization rate values for systems with HAPs (O184 and O127) were at an average level (approximately 6 W/g) and were higher than for APO mixtures, but the time needed to achieve *R_p_^max^* was the longest (over 60 s). Photopolymerization with HAPs occurred much faster and in a shorter time at a wavelength of 365 nm and low irradiance (Figure 4a). The manufacturer’s information shows that this type of photoinitiator does not absorb radiation at this wavelength; their λ_max_ is approximately 243 and 330 nm (Table 1). This may be due to the presence of P-telogen in the system. The molar concentration of DMPh is higher than that of the photoinitiator. There may have been a shift in the absorbance of the HAP + DMPh photoinitiating system towards longer wavelengths. In turn, the tested photoinitiators from the APO group or their blends showed the maximum absorption at approximately 370 nm. Hence, the conclusion is that they are better suited to the source of UV radiation. Additionally, they can be used in formulations containing monomers that are strong triplet quenchers, such as styrene. Regardless of the UV range and UV irradiance, in each tested exposure variant, the highest reaction rates were achieved by systems with O819. The only exception was the system in which the UV irradiance was high and the UV range was wide (Figure 4c), whereby the sample with O184 achieved the highest *R_p_*. Nevertheless, this was due to the molar concentration of this photoinitiator being the highest. An additional feature of O819 is the generation of four radicals per photoinitiator molecule. A trimethylbenzoyl radical and trimethylbenzoylphosphinyl radical are formed from the primary scission, both of which are active. The latter radical can undergo further scission, producing a second trimethylbenzoyl radical and a phosphinyl radical, making them very fast and efficient photoinitiators. This is also confirmed by the photoinitiation index results shown in Figure 5a.

Interestingly, in the case of all initiating systems used in the process (PI + DMPh), regardless of the type of photoinitiator and at the same low value of I_o_ (90 mW/cm^2^), the *I_p_* values were higher when irradiated at 365 nm than in a wide UV range. The *I_p_* increased significantly with increasing I_0_ (500 mW/cm^2^, Figure 5b). Among acylphosphine oxides, the initiation efficiency was as follows: O819 < OTPO < OTPOL. This is consistent with the literature data. However, among the APO blends, in a wide UV range and regardless of the UV irradiance, the initiation efficiencies with O2100 (a mixture of two acylphosphine oxides) and BL750 (a mixture of three oxides) were similar. At 365 nm, O2100 performed better. The kinetic studies of the photopolymerization/phototelomerization processes of the coating composition clearly indicate the higher reactivity of systems with APOs (especially O819) than HAPs. However, APO blends give worse results than single APOs.

### 3.2. Comparison of UV LEDs and Medium-Pressure Mercury Lamp Used to Cure Coatings

**Figure 6:**
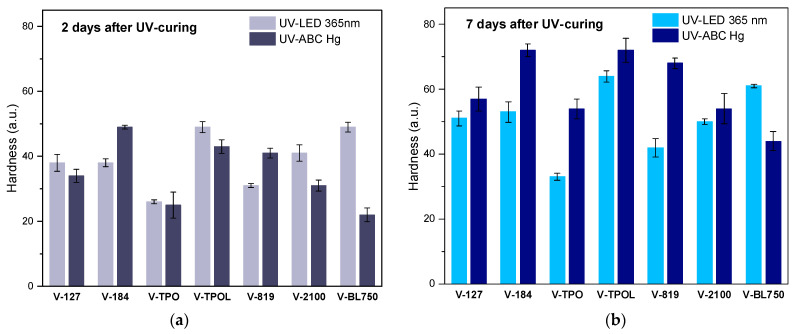
Hardness of the UV-cured varnish coatings cured using UV LEDs or medium-pressure mercury lamp (UV dose of 6 J/cm^2^) and tested after 2 (**a**) and 7 (**b**) days.

As mentioned above, in the present study, the influence of the UV radiation type in the photocrosslinking process on the hardness of the coatings was tested. UV LEDs (365 nm) or a medium-pressure mercury lamp that emitted UV-ABC radiation (200–420 nm) were used. The effectiveness of the photocrosslinking process was checked using the same UV dose for UV light sources (6 J/cm^2^). The UV dose (J/cm^2^) is the total UV energy applied on the coating surface and is released from the lamps during exposure. The dependence of the varnish hardness on the type of photoinitiator and the type of UV-radiating source is shown in Figure 6. Hardness testing was carried out 2 and 7 days after irradiation. Although it is generally believed that radical photopolymerization (as in this case) ends when the light source is switched off, in fact, only the generation of radicals ends, and the radicals that have already formed, in the form of macroradicals, can also participate in the crosslinking process in the dark.

**Figure 7:**
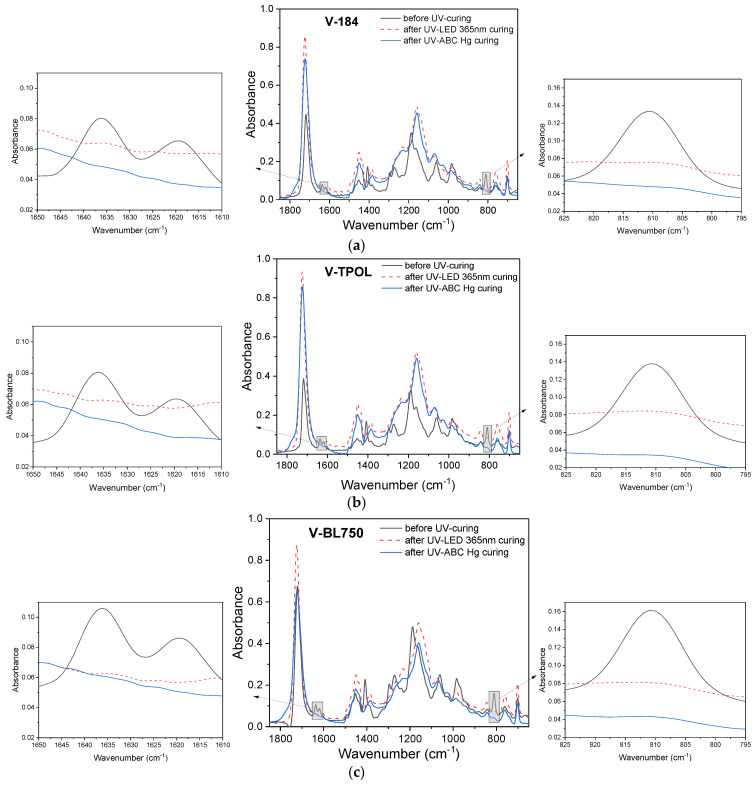
FTIR spectra for the varnish compositions (before UV curing) and for UV-cured varnish coatings 7 days after UV curing process using UV LEDs or a UV medium-pressure mercury lamp (UV dose of 6 J/cm^2^): (**a**) V-184, (**b**) V-TPOL, (**c**) V-BL750.

This can be seen by comparing the varnish hardness results after 2 and 7 days (Figure 6a,b). The latter are higher. The hardness values of the coatings depend mainly on the density of the polymer network. In the studies described here, no multifunctional crosslinking monomer (e.g., PETIA) was used at the photocrosslinking stage to increase the density of the polymer network and improve the hardness of the coatings. This action was intentional. Therefore, the obtained hardened coatings were characterized by relatively low hardness after two days (from approximately 30 to 50 a.u.). The V-O184, V- OTPOL, and V-BL750 coatings were characterized by the highest hardness values, but, in the case of V-184, exposed to the UV-ABC lamp, higher hardness was achieved (52 a.u.) than when using UV LEDs (39 a.u.). In the case of V-TPOL, the hardness values were similar regardless of the radiation source (48–51 a.u.), and the V-BL750 coating was much harder after crosslinking with a UV LED lamp than with UV-ABC (53 and 28 a.u., respectively). Although, after 7 days after exposure, an increase in hardness was observed for each coating, V-184 and V-TPOL were harder after exposure to a UV-ABC lamp (approximately 74 a.u.), as well as V-BL750 after exposure to UV LEDs. The results were slightly different for these three coatings, but, in each case, the FTIR tests revealed the disappearance of bands characteristic of double bonds (Figure 7).

**Figure 8:**
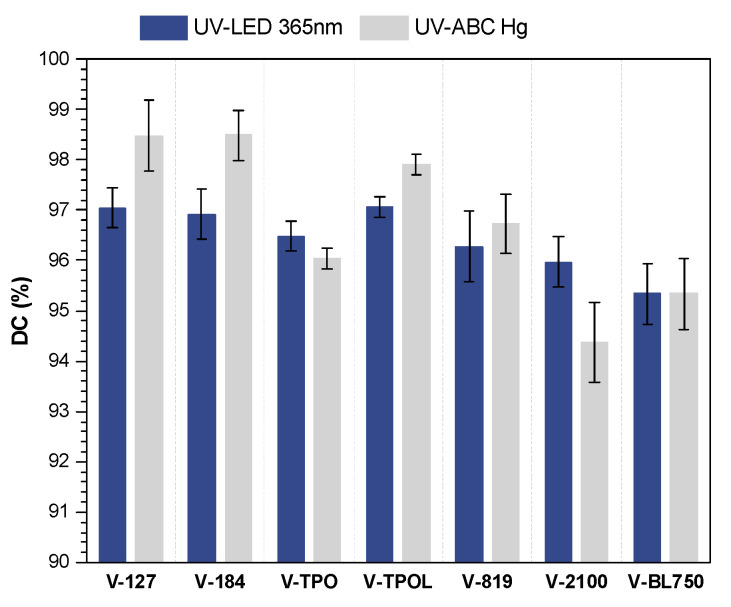
Conversion of double bonds (DC_C=C_) for the UV-cured varnish coatings (6 J/cm^2^).

The conversion of double bonds seven days after exposure depending on the type of UV radiation source and the type of photoinitiator is shown in Figure 8. The DC values were higher than 94%. The highest results (ca. 98%) were obtained in the case of coatings with HAP photoinitiators, because PIs that have strong absorption in the short-wave UV range give good surface curing. Interestingly, even after crosslinking using a UV LED lamp, the DC for these samples (V-127 and V-184) was the highest (similar to V-TPOL, 97%).

### 3.3. Comparison of Different UV Doses

**Figure 9:**
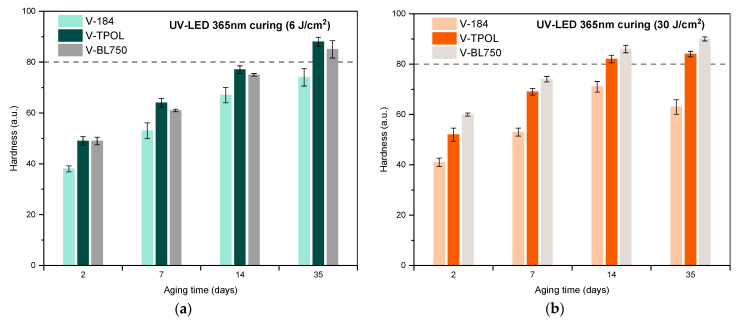
Hardness of the coatings V-184, V-TPOL, and V-BL750 cured using UV LEDs with a dose of 6 J/cm^2^ (**a**) or 30 J/cm^2^ (**b**) (tests were performed 2, 7, 14 and 35 days after the UV curing process).

The above studies showed that even low-energy UV radiation emitted from the LED lamp at 365 nm caused the hardening of the coatings. The best results were obtained for the V-TPOL and V-BL750 systems. It should also be mentioned that no oxygen inhibition effect was observed even with the use of APOs. It is known from the literature that APOs give excellent depth curing but relatively poor surface curing, since the phosphinyl radicals are very sensitive to oxygen. In this study, an organophosphate compound, i.e., DMPh, was also present in the photoreactive coating composition. DMPh is composed of di-esters of phosphorous acid. As shown by NMR studies, DMPh is a weak chain transfer agent (weak telogen) because it is incorporated at only 30% into the oligomer structure. However, it is known from the literature that phosphoric acid(III) esters are good antioxidants and may react with peroxygen radicals [35]. For this reason, even at a low UV dose and low UV irradiance (90 mW/cm^2^), no oxygen inhibition phenomenon was observed (the coatings were dry). This was also confirmed by FTIR tests (high DC values). In the next step, the hardness results of coatings cured with a UV LED lamp were compared, but at a higher UV dose (30 J/cm^2^). The results are presented in Figure 9. Moreover, the influence of the aging time of the prepared coatings was analyzed.

**Figure 10:**
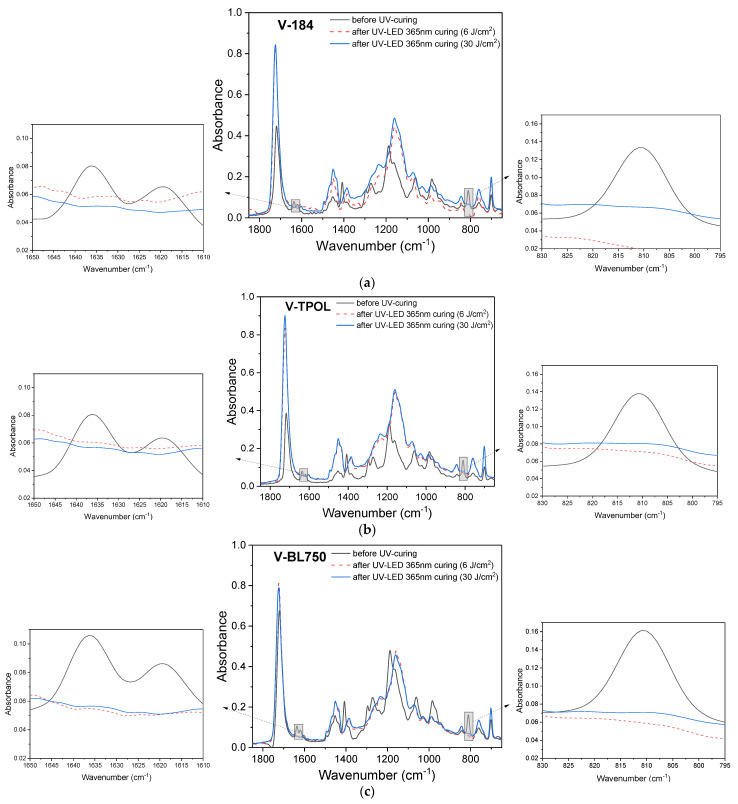
FTIR spectra for the varnish compositions (before UV curing) and for UV-cured varnish coatings 35 days after UV curing process using UV LEDs or a UV medium-pressure mercury lamp (UV dose of 6 or 30 J/cm^2^): (**a**) V-184, (**b**) V-TPOL, (**c**) V-BL750.

As can be seen, the hardness values of the coatings increased by almost twice as they aged from 2 to 35 days—for example, from 50 a.u. for V-TPOL and up to 90 a.u. V-BL750. When a low dose of UV LED at 365 nm was applied, better hardness results were achieved each time by V-TPOL. On the other hand, the photoinitiator BL750 proved to be better at higher UV doses (30 J/cm^2^), as the V-BL750 coating had already achieved the desired hardness value of 80 a.u. after 14 days of aging. However, the lowest hardness values were noted for varnishes with O184. FTIR studies showed the almost complete disappearance of the 812 cm^−1^ and 1635 cm^−1^ characteristic bands in the prepared coatings (Figure 10).

**Figure 11:**
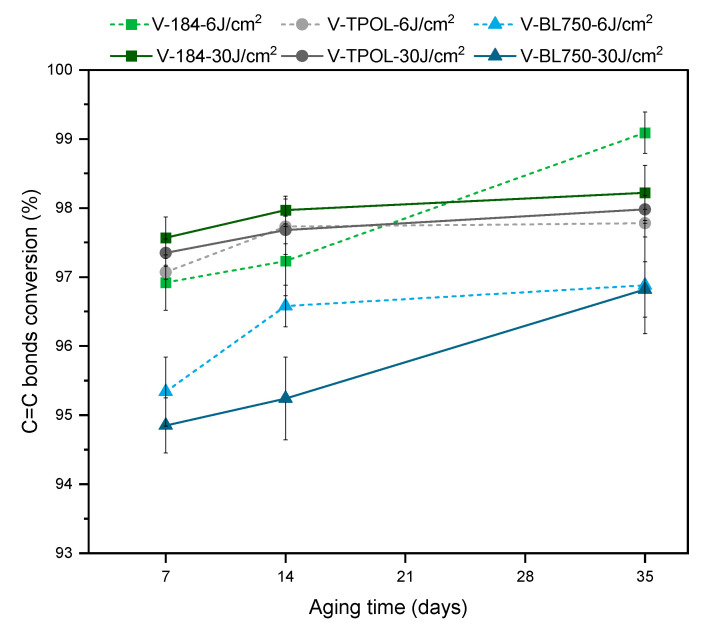
Conversion of double bonds (C=C bonds) in coatings: V-184, V-TPOL, and V-BL750 (tests were performed 7, 14 and 35 days after the UV curing process using UV LEDs with a UV dose of 6 J/cm^2^ or 30 J/cm^2^).

The conversion of double bonds in varnishes V-184, V-TPOL, and V-BL750 after aging is shown in Figure 11. All DC values were increased over time. In addition, in the systems with O184 and OTPOL, a higher UV LED dose caused the greater conversion of C=C bonds (approximately 0.5%). 

## 4. Conclusions

In this study, the UV curing process of coatings based on (meth)acrylic/styrene telomers with terminal P-atoms (prepared via a UV phototelomerization process) was analyzed. The influence of the type of photoinitiator (HAPs, APOs, or APO blends), as well as the UV radiation source (medium-pressure mercury lamp or UV LEDs), was tested. The UV curing kinetics and hardness of the varnishes were presented. The main conclusions are as follows:

- Photopolymerization/phototelomerization processes during the UV curing of the coating formulations occurred much faster at high UV irradiance (conditions such as the UV curing process realized with a UV-ABC-type medium-pressure mercury lamp). Moreover, the kinetics of these processes indicated the higher reactivity of systems with APOs than HAPs (regardless of the incident light intensity).
- Among acylphosphine oxides, the initiation efficiency is as follows: O819 < OTPO < OTPOL.
- The kinetic results did not correspond with the hardness values of the coatings. The APO systems with the lowest reactivity (V-TPOL) allowed the preparation of the hardest varnishes.
- The formulations with TPOL were suitable both for curing with UV LED at 365 nm and a UV-ABC-type medium-pressure mercury lamp (hardness values were similar).
- The presence of phosphoric acid diesters, e.g., dimethyl phosphite, has a beneficial effect on the photocrosslinking process at a low UV dose and low UV irradiation, because DMPh acts as an antioxidant, which allows for effective crosslinking even when using APO-type photoinitiators.

Coating compositions based on phosphorus (meth)acrylate telomeres and containing free monomers and dimethyl phosphite (as a telogen and antioxidant) can be used even without the use of a crosslinking monomer to produce relatively hard varnishes. It has been shown that the UV crosslinking process is possible using low-energy radiation sources and photoinitiators from the group of acylphosphine oxides, without the effect of oxygen inhibition in the surface layer. Due to the presence of hydroxyl groups in the coating (derived from 2-hydroxyethyl acrylate), they are characterized by good adhesion to glass and metal (as shown in [32]). Additionally, a positive influence is exerted by P=O groups (from telogen), which is related to the formation of hydrogen bonds between the P=O groups and the HO-Si groups from the surface.

## Figures and Tables

**Table 1:** Reaction mixture used for the preparation of the P-telomer syrup (P-TS).

Monomers (wt%)				P-Telogen (wt. parts)	PI (wt. parts)
BA	MMA	HEA	STY	DMPh	O819
40	35	15	10	1.65	0.75

**Table 2:** The compositions of the UV-curable varnishes.

Varnish Symbol	Components			
	P-TS (wt. parts)	Photoinitiator		
		Symbol	Dose (wt. parts)	Dose (mol) *
V-127	100	O127	1	0.00294
V-184		O184		0.00489
V-TPO		OTPO		0.00287
V-TPOL		OTPOL		0.00316
V-819		O819		0.00238
V-2100		O2100		0.00243
V-BL750		OBL750		0.00291

* mole/100 wt. parts of P-TS.

**Table 3:** Properties of the P-telomer syrup and dry P-telomers.

SC (%) *	η (Pa·s)	M_n_ (g/mol)	M_w_ (g/mol)	PDI (a.u.)	Monomer/Telogen Conversion (%) *				
					BA	MMA	HEA	STY	DMPh
47	1.2	14,300	48,300	3.4	27	64	30	77	30

*—according to NMR data.

## Data Availability

The data presented in this study are available on request from the corresponding author.

## References

[1] Sheel Wali A., Kumar S., Khan D. (2023). A Review on Recent Development and Application of Radiation Curing. Mater. Today Proc..

[2] Fu J., Wang L., Yu H., Haroon M., Haq F., Shi W., Wu B., Wang L. (2019). Research Progress of UV-Curable Polyurethane Acrylate-Based Hardening Coatings. Prog. Org. Coat..

[3] Javadi A., Mehr H.S., Sobani M., Soucek M.D. (2016). Cure-on-Command Technology: A Review of the Current State of the Art. Prog. Org. Coat..

[4] Glöckner P., Jung T., Struck S., Studer K. (2008). Radiation Curing. Coatings and Printing Inks: Technical Basics, Applications and Trouble Shooting.

[5] Lommes J., Patzelt G., Stenzel V. (2023). UV-Curable Polyimide/Layered Silicate Films with Improved Barrier Properties for the Protection of Semiconductor Chips. Microelectron. Eng..

[6] Zareanshahraki F., Asemani H.R., Skuza J., Mannari V. (2020). Synthesis of Non-Isocyanate Polyurethanes and Their Application in Radiation-Curable Aerospace Coatings. Prog. Org. Coat..

[7] Vijayan S.P., Aparna S., Sahoo S.K. (2023). Effect of Beeswax on Hydrophobicity, Moisture Resistance and Transparency of UV Curable Linseed Oil Based Coating for Compostable Paper Packaging. Ind. Crops Prod..

[8] Sun Y., Xu J., Long L., Gong J., Chen M., Liu R. (2023). A Novel Self-Wrinkled Polyurethane-Acrylate Wood Coating with Self-Matting, Anti-Fingerprint Performance and Skin-Tactile Feeling via Excimer Lamp/UV Curing. RSC Adv..

[9] Liu H., Liu X., Rao Y., Shen X., Tang Z., Chen H. (2023). Facile Fabrication of Robust and Universal UV-Curable Polyurethane Composite Coatings with Antibacterial Properties. Polym. Eng. Sci..

[10] Tsujimura Y., Fukuyama T., Hamano N., Iwashita H., Watanabe M., Ino S. (2023). The Stain Resistant Effect of an Ultraviolet Curable Coating Material on Denture Base Resin. Dent. Mater. J..

[11] Li T., Lin F., Ji M., Huang B., Cheng W., Shi L., Xia M., Wang L. (2021). Development and Measurement of a 365 NM UV LED Irradiance Source. Meas. Sens..

[12] Borysiuk P., Derda M., Auriga R., Boruszewski P., Monder S., Dobrowolska E., Zbieć M. (2015). Comparison of Selected Properties of Varnish Coatings Curing with the Use of UV and UV-LED Approach. Annals of Warsaw University of Life Sciences—SGGW Forestry and Wood Technology.

[13] Jašúrek B., Vališ J., Syrový T., Vladić G. (2022). Development of New UV LED Curable Inkjet Varnishes. Proceedings of the 11th International Symposium Graphic Engineering and Design.

[14] Commission Delegated Directive (EU) 2022/279 of 13 December 2021 Amending, for the Purposes of Adapting to Scientific and Technical Progress, Annex III to Directive 2011/65/EU of the European Parliament and of the Council as Regards an Exemption for the Use of Mercury in Other Discharge Lamps for Special Purposes. https://eur-lex.europa.eu.

[15] Muramoto Y., Kimura M., Nouda S. (2014). Development and Future of Ultraviolet Light-Emitting Diodes: UV-LED Will Replace the UV Lamp. Semicond. Sci. Technol..

[16] Landry V., Blanchet P., Boivin G., Bouffard J.-F., Vlad M. (2015). UV-LED Curing Efficiency of Wood Coatings. Coatings.

[17] Peng W., Su D. Application of LED Lighting in Ultraviolet Curing with Circular Economy Benefits. Proceedings of the 26th International Conference on Automation and Computing (ICAC).

[18] Schwalm R. (2007). UV Coatings: Basic, Recent Developments and New Application.

[19] Ghazali S.K., Adrus N., Majid R.A., Ali F., Jamaluddin J. (2021). UV-LED as a New Emerging Tool for Curable Polyurethane Acrylate Hydrophobic Coating. Polymers.

[20] El Asri Z., Chougrani K., Negrell-Guirao C., David G., Boutevin B., Loubat C. (2008). An Efficient Process for Synthesizing and Hydrolyzing a Phosphonated Methacrylate: Investigation of the Adhesive and Anticorrosive Properties. J. Polym. Sci. A Polym. Chem..

[21] Ma G.Y., Wang C.J., Du C.B., Li X., Wang X.R. (2022). A Corrosion-Resistance Waterborne Polyacrylate Coatings Based on Novel Phosphate Esters Polymeric Surfactant. J. Appl. Polym. Sci..

[22] Ozman E., Dizman C., Birtane H., Kahraman M.V. (2023). Novel Bio-Based Phosphorous-Containing UV-Curable Flame-Retardant Coatings. J. Coat. Technol. Res..

[23] Karkosh Z.S.A., Hussein B.M.A., Al-Wattar W.M.A. (2018). Effect of Phosphoric Containing and Varnish-Coated Groups on Candida Albicans Adhesion and Porosity of Heat Cure Acrylic Denture Base Material. Biomed. Pharmacol. J..

[24] Monge S., Canniccioni B., David G., Robin J.-J., Monge S., David G. (2014). Polymerization of Phosphorus-Containing (Meth)Acrylate Monomers. Phosphorus-Based Polymers: From Synthesis to Applications.

[25] Mukumoto K., Zhong M., Matyjaszewski K. (2014). Atom Transfer Radical Polymerization of Dimethyl (Methacryloyloxymethyl) Phosphonate. Eur. Polym. J..

[26] Hajiali F., Tajbakhsh S., Marić M. (2020). Thermal Characteristics and Flame Retardance Behavior of Phosphoric Acid-Containing Poly(Methacrylates) Synthesized by RAFT Polymerization. Mater. Today Commun..

[27] Solimando X., Kennedy E., David G., Champagne P., Cunningham M.F. (2020). Phosphorus-Containing Polymers Synthesised via Nitroxide-Mediated Polymerisation and Their Grafting on Chitosan by Grafting to and Grafting from Approaches. Polym. Chem..

[28] Chung I. (2000). Monte Carlo Simulation of Free Radical Telomerization. Polymer.

[29] Duc M., Boutevin B., Ameduri B. (2021). Unexpected Radical Telomerisation of Vinylidene Fluoride with 2-Mercaptoethanol. Molecules.

[30] Starks C.M. (1974). Free Radical Telomerization.

[31] Kraśkiewicz A., Kowalczyk A. (2022). Phosphorus-Containing Telomers as UV-Curable Binders of Solvent-Free Varnish Coatings. Materials.

[32] Kraśkiewicz A., Kowalczyk A., Kowalczyk K., Markowska-Szczupak A., Idzik T., Sośnicki J. (2023). Anticorrosive and antimicrobial efficiency of photopolymerizable phosphorus (meth)acrylate oligomers-based coating materials. Prog. Org. Coat..

[33] PN-EN ISO 1522 Standard. https://industrialphysics.com/standards/iso-1522/.

[34] Herrera-González A.M., Caldera-Villalobos M., Pérez-Mondragón A.A., Cuevas-Suárez C.E., González-López J.A. (2019). Analysis of Double Bond Conversion of Photopolymerizable Monomers by FTIR-ATR Spectroscopy. J. Chem. Educ..

[35] Pączkowski J. (2003). Photochemistry of Polymers. Theory and Application.

